# Horizontal gene transfer is not a hallmark of the human genome

**DOI:** 10.1186/s13059-017-1214-2

**Published:** 2017-05-08

**Authors:** Steven L. Salzberg

**Affiliations:** Departments of Biomedical Engineering, Computer Science and Biostatistics, Center for Computational Biology, McKusick-Nathans Institute of Genetic Medicine, Johns Hopkins University, Baltimore, MD USA

## Abstract

Crisp et al. recently reported that 145 human genes have been horizontally transferred from distant species. Here, I re-analyze those genes listed by Crisp et al. as having the highest certainty of having been horizontally transferred, as well as 17 further genes from the 2001 human genome article, and find little or no evidence to support claims of horizontal gene transfer (HGT).

Please see related Research article: https://genomebiology.biomedcentral.com/articles/10.1186/s13059-015-0607-3

**Electronic supplementary material:**

The online version of this article (doi:10.1186/s13059-017-1214-2) contains supplementary material, which is available to authorized users.

## Introduction

A recent study by Crisp et al. [1] re-examined a claim, originally made in the landmark 2001 human genome paper, that bacteria had horizontally transferred 223 genes into a vertebrate ancestor of humans [2]. That claim was refuted soon after the original report [3, 4]. Using an alignment-based scoring scheme, the study by Crisp et al. [1] reported that 145 human genes, including 17 of those from the 2001 study, had been horizontally transferred from distant species. Here, I describe a re-analysis of these 17 genes and of the 28 highest-confidence genes newly claimed by Crisp et al. [1] to have been horizontally transferred, taking a more skeptical perspective, and find little or no evidence to support claims of horizontal gene transfer (HGT).

Hundreds of eukaryotic genomes and thousands of bacterial genomes have been sequenced in the 15 years since the human genome was published. In their recent report, Crisp et al. [1] argue that, with the availability of this far larger collection of genomes, the likelihood of false HGT findings that are actually the result of gene loss is now greatly reduced. Their reanalysis, which was based on a combination of BLAST searches and phylogenetic trees, identified hundreds of “foreign” genes in animals; this led them to claim that HGT “has occurred on a previously unsuspected scale in metazoans” and that it is a significant factor in animal evolution.

In this study, I re-examined the claims of Crisp et al. [1] focusing on the human genes. Instead of using a large-scale, automated analysis, which by its very nature could enrich the results for artifactual findings, I looked at each human gene individually to determine whether the evidence is sufficient to support the conclusion that HGT occurred. An important principal here is that extraordinary claims require extraordinary evidence: there is no doubt that the vast majority of human genes owe their presence in the human genome to the normal process of inheritance by vertical descent. Thus, if other, more mundane processes can explain the alignments of a human gene sequence, these explanations are far more likely than HGT.

## Results

For my re-analysis, I re-aligned the 17 human genes that were originally reported as having undergone bacterial-vertebrate transfer (BVT), a finding that has been rejected by our work [3] and that of others [4, 5], but re-claimed by Crisp et al. [1] (Table 1). I found that the evidence does not support HGT for any of them. (One important point worth noting here is that Crisp et al. listed some of these genes as “confirmed” by Salzberg et al. [3]. This was not the case; our previous study invalidated most of the previously claimed HGT events, but was not able to dismiss all of them. Our study made it clear that we did not consider the presence of the remaining genes to be the result of HGT events.) Crisp et al. [1] reported a total of 145 human genes that they claimed to be the result of HGT; 39 of these are labeled in their highest confidence group, class A. Of these 39, seven are included in the first group of 17, leaving 32 newly claimed HGT events. I examined these 32 class A genes (Table 2) and again find no evidence for HGT. A detailed, gene-by-gene description of these analyses can be found in Additional file 1 and the sequences of the genes in Tables 1 and 2 can be found in Additional file 2.

**Table 1:** Re-analysis of 17 human genes claimed as examples of horizontal gene transfer

Gene group number	Ensembl gene ID	Gene name	Best metazoan, non-chordate match		Best non-metazoan match		Explanation	
			Species	Bit score	Species	Bit score	Crisp et al.	This study
1	ENSG00000166743, ENSG00000183747, ENSG00000005187, ENSG00000183549	Acyl-CoA synthetase medium-chain family members 1, 2A, 3, and 5	*Lingula anatina* or *Aplysia californica*	686	*Desulfotomaculum thermocisternum* or *D. kuznetsovii*	668	Bacterial HGT	No HGT
2	ENSG00000047457	Ceruloplasmin (ferroxidase)	*Exaiptasia pallida* (sea anemone)	898	*Klebsormidium flaccidum* (alga)	543	Plant HGT	No HGT
3	ENSG00000107618, ENSG00000265203	Retinol-binding protein 3	None		*Stackebrandtia nassauensis*	152	Bacterial HGT	Gene loss
4	ENSG00000181019, ENSG00000124588	NAD(P)H dehydrogenase, quinone 1 and 2	*L. anatina* or *Capitella teleta*	245	*Sinorhizobium fredii*	204	Bacterial HGT	No HGT
5	ENSG00000132744	Aspartoacylase (aminocyclase) 3	*Saccoglossus kowalevskii*	262	*Oscillatoria* sp. PCC 10802	249	Bacterial HGT	No HGT
6	ENSG00000170961, ENSG00000105509, ENSG00000103044	Hyaluronan synthase 1, 2, and 3	*Papilio xuthus*	78	*Lichtheimia ramosa*	333	Fungal HGT	Rate variation
7	ENSG00000175806	Methionine sulfoxide reductase A	*Lottia gigantea*	302	*Pleurocapsa* sp. PCC 7319	306	Bacterial HGT	No HGT
8	ENSG00000095596	Cytochrome P450, family 26, subfamily A, polypeptide 1	*C. teleta*	396	*Geitlerinema* sp. PCC 7407	302	Bacterial HGT	No HGT
9	ENSG00000113790	Enoyl-CoA, hydratase/3-hydroxyacyl CoA dehydrogenase	*L. anatina*	645	*Capsaspora owczarzaki*	582	Protist HGT	No HGT
10	ENSG00000166532	Ribosomal modification protein rimK-like family member B	*A. californica*	143	*Kitasatospora cheerisanensis*	194	Archaeal HGT	Rate variation
11	ENSG00000172508	Carnosine synthase 1	*Crassostrea gigas*	614	*Perkinsus marinus*	151	Protist HGT	No HGT

Columns 5 and 7 contain the bitscores of the best BLAST alignment to a gene from the species in columns 4 and 6. *Gene group number* (column 1) refers to the number used in the main text

**Table 2:** Re-analysis of genes claimed to be newly discovered human horizontal gene transfers (HGT) in the Crisp et al. [1] study

Gene ID	Gene name	Results from Crisp et al. [1] study				Results from this study			
		HGT index	Source HGT taxon	Best non-chordate metazoan match	Bit score	Best non-chordate metazoan match	Matching protein ID	Bit score	New HGT index
ENSG00000070269	C14orf101	337	Protist	B4LPG6	37	*Lingula anatina*	XP_013409033	566	–192
ENSG00000102805	Ceroid-lipofuscinosis, neuronal 5	84	Protist	E3MZU2	39	*Lottia gigantea*	XP_009059770	338	–215
ENSG00000116721	PRAME family member 1	72	Protist^a^	A8MVS2	44	No hits			
ENSG00000157358	PRAME family member 15	45	Protist^a^	F1SUY5	89	No hits			
ENSG00000232423	PRAME family member 6	45	Protist^a^	F1SUY5	92	No hits			
ENSG00000117115, ENSG00000142619, ENSG00000142623, ENSG00000159339	Protein-arginine deiminase, types 2,3,1,5	464	Bacteria	B4JS81	45	*Priapulus caudatus*	XP_014670176	560	–51
ENSG00000125458	5′,3′-nucleotidase, cytosolic	135	Bacteria	E3LP71	39	*Saccoglossus kowalevskii*	XP_006825056	222	–48
ENSG00000205309	5′,3′-nucleotidase, mitochondrial	108	Bacteria	B5DJB4	41	*L. anatina*	XP_013404549	237	–88
ENSG00000133561, ENSG00000133574, ENSG00000179144, ENSG00000196329, ENSG00000213203	GTPase, IMAP family members 6, 4, 7, 5, and 1	51	Plant	F1QNI4	94	*Crassostrea gigas*	XP_011437597	208	–63
ENSG00000136153	LIM domain 7	47	Protist	H9JLZ2	86	*S. kowalevskii*	XP_006813620	268	–135
ENSG00000136830	Family with sequence similarity 129, member B	74	Protist	G6DB46	44	*Acropora digitifera*	XP_015774462	115	3
ENSG00000140718	Fat mass and obesity associated	97	Plant	C9J4C3	92	*S. kowalevskii*	ALR88588	342	–153
ENSG00000148288	Globoside alpha-1,3-N-acetylgalactosaminyltransferase 1	91	Bacteria	A8QE87	39	*Cimex lectularis*	XP_014240367.1	37	93
ENSG00000154122	Ankylosis, progressive homolog (mouse)	129	Protist	F5GXN7	90	*S. kowalevskii*	XP_006818212	388	–169
ENSG00000172757	Cofilin 1	31	Fungi	B3S0K8	80	*Helobdella robusta*	XP_009011217	108	3
ENSG00000175573	Chromosome 11 open reading frame 68	60	Fungi	D6WQ69	48	*S. kowalevskii*	XP_002740403.1	157	–49
ENSG00000177181	Ribosomal modification protein rimK-like family member A	74	Archaea	E1GHX3	52	*Aplysia californica*	XP_012936156	124	2
ENSG00000212907	Mitochondrially encoded NADH dehydrogenase 4 L	69	Protist	P15554	68	*Xenoturbella bocki*	YP_850984	67	71
ENSG00000216937	Coiled-coil domain containing protein 7	48	Protist	A7SDV0	64	No hits			
ENSG00000242265	Retrotransposon-derived protein PEG10	52	Fungi	G0MWG3	49	No hits			
ENSG00000256062	ABO blood group (Histo-blood group ABO system transferase)	84	Bacteria	H3INK7	37	*S. kowalevskii*	XM_006825840	218	–97

The HGT index, defined by Crisp et al. [1] as the difference in the best bitscore of a BLAST match to a non-metazoan and a metazoan species, is shown along with the bitscore of the best metazoan match. The best metazoan match excluded any matches to the phylum Chordata for these human genes. All of the genes in this table were reported by Crisp et al. [1] as high-confidence (class A) HGT. The recomputed HGT index (last column) is computed by subtracting the bitscore of best non-metazoan found by Crisp et al. [1] from that of the best non-chordate metazoans found by the new searches reported here. “No hits” means that no significant alignments were found to any non-chordate metazoans

^a^For PRAME family members 1, 6, and 15, the protist alignment found by Crisp et al. [1] is a false positive caused by contamination. See main text for details

Of the 17 genes from the original human genome paper that Crisp et al. [1] claim are true examples of HGT, my analysis finds that 12 genes fail to pass the authors' own BLAST-based test for HGT, because their closest metazoan match has a bitscore that is greater than the best non-metazoan match (Table 1). Of the 28 genes representing new claims of HGT (Table 2), 26 fail the initial screen for HGT candidates, either because they fail the original BLAST bitscore test, because they represent contaminants in draft genomes, or because they are known mitochondrial or retrotransposed genes. The remaining seven genes (five from Table 1 and two from Table 2) include three close paralogs (HAS1–3) and thus represent four hypothesized HGT events. A combination of gene loss and evolutionary rate variation is more than adequate to explain these genes: among other reasons, the alignments and bitscores are the result of screening more than 20,000 human genes, and one might expect a few genes from this large set to be lost (or to have evolved slightly more rapidly) in the non-chordate genomes.

One reason that better BLAST results were found in the current study could well be that this study used data from May 2016, whereas Crisp et al.'s study used data from January 2013. A large number of additional genomes have been deposited in public archives during the three years between the two analyses. These species were not available to the previous study and thus the orthologous genes from these taxa were missed. Insofar as this explanation is correct, it strengthens the argument for gene loss as the explanation for the (very few) human genes that still have better BLAST matches in non-metazoans than in non-chordate metazoans.

Another factor is that because only non-chordates are considered, the alignments and bitscores between a human gene and these very distant relatives are necessarily quite weak. This distant relationship makes it more likely that some genes will not be found simply because the sequence has diverged too much for a pairwise alignment to detect it.

This study focuses only on human genes, but recent claims of high levels of HGT in other animals have also been reported. The most dramatic claim was the recent report that up to one-sixth of the genes in the tardigrade (*Hypsibius dujardini*) had been laterally transferred from other species [6], but that claim was quickly shown to be a false result due primarily to contamination of the genome assembly [7]. In Crisp et al. [1], contamination seems to be a likely explanation for the three human genes (PRAME family members 1, 6, and 15) reported as high-confidence HGT events, and a closer scrutiny of other automatically identified HGT candidates might reveal other cases. (Contamination has been reported to create false signals of HGT as far back as 2002 [8].) My re-examination here suggests that HGT is very rare rather than widespread in vertebrate genomes, and that every hypothesized HGT event needs to be subjected to careful scrutiny.

As we wrote in 2001 [3], “the argument for lateral gene transfer is essentially a statistical one, necessarily so because of the inherent impossibility of observing events that may have occurred in the distant past”. When searching a large set of genes against an even larger database, one must recognize that such large-scale, automated searches will inevitably find unusual results that include genes that were lost or evolved more rapidly in multiple lineages. Because HGT is such an unlikely event, the results of automated searches should be subjected to individual, close scrutiny with an eye toward explaining them through more mundane processes before concluding that these anomalies represent novel biological discoveries. As demonstrated here, a re-analysis using the latest genome databases shows that other than the well-known mitochondrial genome transfer and retrovirus-mediated events, no genes have been horizontally transferred into the human genome.

## Methods

Ensembl identifiers for all genes proposed as examples of HGT were obtained from Crisp et al. [1] and validated by retrieving them from the Ensembl database (www.ensembl.org). Genomes and protein sequences were obtained from the National Center for Biotechnology Information (NCBI; www.ncbi.nlm.nih.gov) and UniProt (www.uniprot.org). Protein sequences were aligned individually using the blastp program and the non-redundant protein database, nr, available through the BLAST server at NCBI (https://blast.ncbi.nih.gov) or for direct download from the same source. To aid analysis, searches were run against the entire database and again with the phylum Chordata (taxon 7711) excluded from the results, which did not affect bitscores.

## Additional files

Additional file 1: Gene-by-gene analysis of evidence for horizontal gene transfer for all genes in Tables 1 and 2. (DOCX 48 kb)

Additional file 2: Protein sequences for Ensembl genes in Tables 1 and 2. (FA 24 kb)

## Abbreviations

BVT: Bacterial-to-vertebrate transfer
HGT: Horizontal gene transfer
NCBI: National Center for Biotechnology Information
