# Supplementary material for: Horizontal gene transfer is not a hallmark of the human genome
Source: Genome Biol. 2017 May 8;18:85. doi: 10.1186/s13059-017-1214-2 (PMC5422933; doi:10.1186/s13059-017-1214-2)
Supplement: Supplementary file 1 — Gene-by-gene analysis of evidence for horizontal gene transfer for all genes in Tables 1 and 2. (DOCX 48 kb) [file 13059_2017_1214_MOESM1_ESM.docx]

**Additional File 1. Horizontal gene transfer is not a hallmark of the human genome.**

Steven L Salzberg

The basis of the claims of horizontal gene transfer (HGT) in Crisp et al. [1] is the HGT index, introduced in 2012 by a subset of the same authors [2]. This index is calculated by aligning a protein to a database using the BLAST program [3], which produces a bitscore that has a standard set of units and thus can be compared to that of other alignments. Higher bitscores correspond to better alignments, and the bitscore can be used directly to compute an E-value, which represents the likelihood that the alignment occurred by chance. The bitscore is thus a measure of how similar two sequences are, which in turn reflects, albeit imperfectly, their evolutionary distance. The HGT index (*h*) defined in Crisp et al. [1] is the difference between the bitscores of the best non-metazoan and the best metazoan matches, *excluding the phylum of the query sequence*. Thus for a human gene, Crisp et al. [1] computed *h* by subtracting the bitscore of the best matching non-Chordata metazoan protein from the bitscore of the best non-metazoan match. If the best non-metazoan match — for example, a bacterial species — had a bitscore at least 30 higher than the best metazoan match (*h* ≥ 30), they considered this gene a candidate for HGT. They built phylogenetic trees to validate these candidates, and rejected genes for which the trees did not support HGT. Trees that supported HGT were required to have a structure in which the human gene, along with other chordate genes, formed a clade within a foreign clade; for example, the chordate gene subtree might be rooted within a bacterial lineage.

Note that for this definition of the HGT index, alignments to chordate genes were excluded. Thus the bitscore for the best metazoan match to a human gene included only non-chordates such as sea urchins, sea anemones, and mussels. By contrast, the method used in the 2001 human genome paper [4] compared the best bacterial match to the best non-vertebrate match, with the non-vertebrates including a plant and a fungus. In that study, genes that were found in bacteria and missing in non-vertebrates were considered as bacteria-to-vertebrate transfers. In the Crisp et al. [1] study, genes were considered as human HGT candidates if they were found in a non-metazoan (bacterial, archaeal, protist or plant) genome but were missing or had a weaker BLAST alignment score to a gene in non-chordate metazoans (i.e., excluding matches to the phylum Chordata). Thus, Crisp et al.'s method searches for genes that were transferred from non-metazoans (not only bacteria but also plants, protists and fungi) into a chordate ancestor of humans. These transfers could have occurred as early as the divergence of chordates from other metazoans, over 500 million years ago [5], or at any more recent point of divergence between the human lineage and other species.

Because the BLAST searches of metazoans were limited to non-chordates, for which relatively few genomes (compared to chordates) have been sequenced, it is possible that some human genes have been lost in these distant relatives. A related concern is that all of these genomes are 'draft' genomes in which significant sections of the genome might be missing, and in which the gene annotation is far from complete. Thus, the failure to find a gene in any non-chordate metazoan species might be due to (i) gene loss, (ii) a gap in the genome, or (iii) missing gene annotation. Any of these provides a more likely explanation for anomalous BLAST results than HGT.

For my re-analysis, I re-aligned the 17 human genes that were originally reported as having undergone bacterial-vertebrate transfer (BVT), a finding that was rejected by our work [6] and that of others [7, 8], but re-claimed by Crisp et al. [1]. Below, I discuss each of these 17 genes, and show that the evidence does not support HGT for any of them. (One important point worth noting here is that Crisp et al. listed some of these genes as 'confirmed' by Salzberg et al. [6]. This was not the case; our previous study invalidated most of the previously claimed HGT events, but was not able to dismiss all of them. Our study made it clear that we did not consider the remaining genes to be the result of HGT events.) Crisp et al. [1] identified a total of 145 human genes that resulted from HGT; 39 of these are labeled in their highest confidence group, class A. Of these 39, seven are included in the first group of 17, leaving 32 newly claimed HGT events. I examined these 32 class A genes and again found no evidence of HGT.

**Re-analysis of 17 previously identified human genes re-claimed as examples of HGT** **{1st level heading}**

Table 1 lists the 17 genes from the original human genome paper’s [4] claim of BVT that Crisp et al. [1] argue were horizontally transferred into the human lineage. Table 1 shows the gene's identifier and name followed by the non-chordate metazoan in which I found the best BLAST match, including its bitscore. It then shows the best non-metazoan match and bitscore found by my searches. The 17 genes fall into 11 groups (because some of them are members of multi-gene families), which I now consider in turn.

***Group 1*, *acyl-CoA synthetase medium-chain family members 1*, *2A*, *3*, *and 5*** **{2nd level heading}**

These four genes are close paralogs, probably the result of internal duplications at some time in the past. Crisp et al. [1] identified family member 3 as an example of HGT from bacteria based on a bacterial match with a bitscore of 637; I found a better match, with a bitscore of 668, to a gene from the bacterium *Desulfotomaculum thermocisternum*. However, while Crisp et al. found the best non-chordate match was to *Nematostella vectensis* (starlet sea anemone), with a bitscore of 473, I found a gene from *Lingula anatina* (tailed mussel, a brachiopod) with a bitscore of 686 (Table 1).

Thus, the best-matching non-chordate actually has a higher bitscore than the best-matching bacterium, meaning that its HGT index *h* would be negative. It therefore fails to satisfy Crisp et al.'s criterion that *h* ≥ 30 and is not an example of HGT.

All four members of this family have similar results: the best bitscore from the bacterial kingdom is lower than that for the best non-chordate. For example, family member 1 has two equally good matches to *L*. *anatina* (tailed mussel) and *Aplysia californica* (sea slug), with bitscores of 532, while its best bacterial match has a lower bitscore of 512.

Further weakening the claim for HGT in this gene family is the fact that these are mitochondrial genes. As explained previously [6], mitochondrial genes do resemble bacterial genes because they derive from the engulfment of the ancestral mitochondrion. Crisp et al.'s HGT events are not supposed to include this evolutionary event, but rather to represent an ongoing process of HGT that occurred after metazoans split from non-metazoans.

Note that if even one member of a gene family was inherited vertically (i.e., not by HGT), then all other members of that family must also have been inherited vertically, because gene families are created by internal gene duplications. Thus none of these four genes should be considered horizontal transfers. Curiously, Crisp et al. [1] initially identified a fifth paralog from this family (member 2B) as having undergone HGT, but excluded it because their own phylogenetic validation step failed to support it. On this evidence, though, all members of this gene family should have been excluded from further consideration as having undergone HGT.

***Group 2*, *ceruloplasmin*** **{2nd level heading}**

This gene was reported by Crisp et al. [1] as an example of HGT from plants to animals, with a best metazoan bitscore of 379 and a best foreign bitscore of 448, from a terrestrial alga. My analysis agreed that the best non-metazoan match was to a plant, *Klebsormidium flaccidum* (a terrestrial alga) with a bitscore of 543, higher than that found by Crisp et al. The best invertebrate match was to *Branchiostoma floridae*, with a bitscore of 984; and excluding chordates, the best metazoan match is a gene from *Exaiptasia pallida* (sea anemone), with a bitscore of 898. Crisp et al. instead found that the best metazoan match was a more-distant alignment to a protein from the sea urchin *Strongylocentrotus purpuratus*.

Therefore, the best metazoan alignment has a higher bitscore than the best non-metazoan alignment for this gene, ruling it out as a candidate for HGT.

***Group 3*, *retinol binding protein 3*** **{2nd level heading}**

Crisp et al. claimed this gene was transferred from bacteria to chordates on the basis of a best bacterial bitscore of 152 and a best score to a non-chordate, *Caenorhabditis elegans*, of 48.9. I found a best bacterial match to *Massilia* sp. LC238 with a bitscore of 172. This gene is widespread in vertebrates but does not appear to be present in non-chordates. It is present, however, in the plant *Ricinus communis* (castor bean) with a bitscore of 122, not much lower than that of the bacterial match.

This finding appears to be an example of gene loss. It does not require a large number of gene loss events to explain it, because the loss is so ancient, possibly near the base of the Opisthokonts, that it may have involved only a few other non-chordate lineages. Alternatively, the gene might have diverged more rapidly in other lineages, so that BLAST searches cannot detect the alignment today. The very ancient divergence of this gene from vertebrates makes either of these two explanations plausible.

***Group 4*, *NAD(P)H dehydrogenase*, *quinone 1 and 2 (NQO1 and NQO2)* {2nd level heading}**

For these genes, Crisp et al. [1] found a best metazoan bitscore of 171 (for the alignment to *C*. *elegans*) and a best bacterial bitscore of 203. For *NQO1*, I found a best bacterial bitscore of 204, to the species *Sinorhizobium fredii*. However, the best metazoan, non-chordate match was *L*. *anatina* (tailed mussel), with a bitscore of 245. For *NQO2*, I found a match to *L*. *anatina* with a bitscore of 230 and an equally good match (bitscore 229) to *Capitella teleta*, a segmented worm from the phylum Annelida. Thus for these genes, the best metazoan match is better than the best bacterial match, and their HGT index is negative, failing to meet the requirement for HGT.

***Group 5*, *aspartoacylase (aminocyclase) 3*** **{2nd level heading}**

Crisp et al. [1] reported that this gene represents HGT from bacteria to human on the basis of a best bacterial bitscore of 202, to *Trichodesmium erythraeum*, and a best metazoan match of 168, to *Nematostella vectensis* (starlet sea anemone). I found the best bacterial match was *Oscillatoria* sp. PCC 10802, with a bitscore of 249. However, a non-chordate metazoan, the acorn worm (*Saccoglossus kowalevskii* (phylum Hemichordata)) has a higher bitscore of 262. Thus, the best non-chordate match is better than the best bacterial match, disqualifying this gene as a candidate for HGT.

***Group 6*, *hyaluronan synthase 1*, *2*, *and 3*** **{2nd level heading}**

Crisp et al. [1] argue that *HAS1–3* represent a newly discovered HGT event from fungi to chordates. The original claim of BVT was debunked in part by showing that a phylogenetic tree did not support it [6]. Crisp et al. [1] agreed that the BVT claim was erroneous, but they reported instead that these genes were transferred from fungi to chordates. In addition to their bitscore data, they show a phylogenetic tree to support their finding.

The best non-metazoan match found by Crisp et al. [1] was to a fungus, with a bitscore of 268. I found similar matches, with bitscores of 322 and 333, to the fungi *Lichtheimia corymbifera* (for *HAS-1*) and *L*. *ramosa* (for *HAS-3*). I did not find matches to vertebrates, but I did find a strong match to *Branchiostoma floridae* (amphioxus), with a bitscore of 686. Crisp et al. reported a best non-chordate metazoan match to *Loa loa* (a filarial nematode) with a bitscore of 57, while I found a slightly better match to *Papilio xuthus* (a butterfly) with a bitscore of 78. The best fungal match is much better than either of these.

This gene family does not appear to be detectable in BLAST searches of the non-chordate metazoans that have been sequenced to date. Because relatively few of these genomes have been sequenced and annotated, a hypothesis of gene loss is plausible. In addition, because these genes are present in amphioxi, which are chordates from a different subphylum, the HGT event must have happened at the very base of the chordate phylogeny, making it a very ancient event. This raises another possible explanation for the BLAST results: rapid evolution in other lineages. Even slightly more rapid evolution of this gene along the non-chordate lineages could make it impossible to detect in the sequences available today.

Nonetheless, many arthropods have a hyaluronan synthase ortholog, which presumably derives from a common ancestor with humans. The hypothesis proposed by Crisp et al. [1] would require that the common ancestor of the human-arthropod genes was lost in the human lineage, to be replaced by a horizontally transferred gene derived from fungi. This pair of events would seem to be much less likely than the simpler explanation that this gene evolved more rapidly in the arthropod lineage and in the non-chordate genomes that have been sequenced. Evolutionary rate variation would also explain the tree shown in Crisp et al. as support for their fungi-to-chordate HGT claim. Thus, either gene loss or evolutionary rate variation are sufficient to explain the inability to find the *HAS1–3* genes in non-chordate genomes.

***Group 7*, *methionine sulfoxide reductase A*** **{2nd level heading}**

Crisp et al. [1] claim that this gene has undergone HGT on the basis of a best bacterial bitscore of 295 and a best non-chordate match to *Trichoplax adhaerens* (the only known member of its phylum, Placozoa) with a bitscore of 159. I found a similar best bacterial match to *Pleurocapsa* sp. PCC 7319, with a bitscore of 306. However, I also found a near-equal match to *Lottia gigantea* (owl limpet) from the phylum Mollusca, with a bitscore of 302. Thus, with an HGT index of 4, this gene is not a candidate for HGT. In addition, the annotation of this protein indicates that it is mitochondrial in origin.

***Group 8*, *cytochrome P450*, *family 26*, *subfamily A*, *polypeptide 1*** **{2nd level heading}**

It is surprising that Crisp et al. [1] reported that only a single member of this large gene family was horizontally transferred while the other members were not. Humans have 18 cytochrome P450 family members encoding 57 genes [9]. As with any gene family, the multiple copies were created through duplications within the genome, not through HGT. Nonetheless, Crisp et al. [1] argue that this gene is an example of HGT from bacteria on the basis of a best non-chordate match with a bitscore of 244, and a best match to bacteria with a bitscore of 290.

I found a similar best bacterial match, to *Geitlerinema* sp. PCC 7407, with a bitscore of 302. However, the best metazoan non-chordate match is to *C*. *teleta* (phylum Annelida) with a bitscore of 396, higher than the best bacterial match. This gene also has slightly weaker matches to *L*. *anatina* and *A*. *californica* (bitscores 392 and 390), as well as to other non-chordates. Thus, this cytochrome P450 gene family member does not appear to have been horizontally transferred from bacteria.

***Group 9*, *enoyl-CoA*, *hydratase/3-hydroxyacyl CoA dehydrogenase*** **{2nd level heading}**

This gene was claimed to have been horizontally transferred from a protist, on the basis of a best metazoan match to *Trichoplax adhaerens* with a bitscore of 479, and a slightly better protist match to *Capsaspora owczarzaki*, with a bitscore of 539. *Capsaspora owczarzaki* is a single-celled eukaryote that is the closest known unicellular relative of the metazoans [10]. I found a similar alignment, with a bitscore of 582.

Among the non-chordate metazoans, I found the best hit to be *L*. *anatina*, with a bitscore of 645, and good hits to other non-chordates (bitscores around 600) including *A*. *california*, *Biomphalaria glabrata*, and *C*. *teleta*. These alignments are consistent with the phylogenetic relationship of these metazoans and the holozoan *Capsaspora*, and thus there is no need to hypothesize that this gene was a product of HGT.

***Group 10*, *ribosomal modification protein rimK-like family member B (also called β-citrylglutamate synthase B)*** **{2nd level heading}**

Crisp et al. [1] reported that this gene had undergone archaeal gene transfer, on the basis of a best metazoan bitscore of 46 for the alignment to *Loa loa*, a filarial nematode, and a best archaeal bitscore of 110, for the alignment to a gene from *Methanosarcina mazei*.

I found the best non-metazoan match was to either of two bacterial species, *Kitasatospora cheerisanensis* and *Streptomyces* sp. XY431, with a bitscore of 194. The best metazoan, non-chordate match is to *A*. *californica* with a bitscore of 143. Thus, although the best metazoan match has a higher score than the best archaeal match, invalidating the claim of archaea-to-vertebrate HGT, the best bacterial match has a higher bitscore. Given the very distant relationship among all of these species, these alignments are probably due to (slightly) more rapid evolution in the *Aplysia* lineage. The HGT hypothesis would require not only horizontal transfer of this gene into chordates, but also loss of the orthologous gene that currently exists in *A*. *californica* and other non-chordates.

***Group 11*, *carnosine synthase 1*** **{2nd level heading}**

Crisp et al. [1] report that this gene has undergone protist-to-chordate HGT. They based this conclusion on a best match to *C*. *elegans* with a bitscore of 57 and a best protist match to *Perkinsus marinus* (a pathogen of oysters) with a bitscore of 151. I found the same match to *P*. *marinus* with a similar bitscore; however, I also found a much better metazoan match, with a bitscore 614, to *Crassostrea gigas* (Pacific oyster) from the phylum Mollusca. (I also found matches to *Octopus bimaculoides* and *L*. *anatina* with much higher scores than the protist match.) Thus, this gene's alignment are consistent with normal vertical inheritance and it fails to meet Crisp et al.'s criteria for HGT.

**Re-analysis of 28 human genes newly identified as HGT**

The highest-confidence HGT events in the Crisp et al. [1] study were labeled as class A, defined as genes with an HGT index of at least 30 that also met two other criteria: a best match to a metazoan with a bitscore <100, and no matches with bitscore above 100 among their ortholog groups. (Ortholog groups for human proteins corresponded to the human-chimp groups from release 5 of OrthoMCL [11].) It is worth re-emphasizing here that the best metazoan match excluded the phylum of the target species; thus for humans, the best metazoan match refers to the best non-chordate, metazoan match. Crisp et al. reported a total of 39 class A HGT genes in humans. Four of these failed their phylogenetic tests, leaving 35 'validated' HGTs. Seven of these were included in the set of 17 genes discussed above. Here, I examine the remaining 28 genes, which Crisp et al. [1] report as newly discovered examples of horizontally acquired genes in humans.

I aligned each gene against all non-chordate genes in GenBank as of May 2016, and recorded the best match as well as its bitscore. I then re-computed the HGT index using the bitscore of the best non-metazoan (bacteria, fungi, protist and so on) found by Crisp et al. [1]. Table 2 summarizes these results. Column 3 of the table shows the HGT index from Crisp et al., which in all cases is >30, as they required. The last column shows the new HGT index based on my re-analysis.

As the table shows, for 21 of the 28 genes, the new HGT index is less than 30, and for 19 of these, it is negative, meaning that the best non-chordate metazoan had a higher bitscore than the presumed source of the HGT. These genes are clearly not examples of HGT.

For three of the remaining seven genes, PRAME family members 1, 6, and 15, the re-analysis revealed that contamination rather than HGT was the explanation for the BLAST alignments. Crisp et al. [1] concluded that all three of these genes were horizontally transferred from protists; in particular, all three had matches to strain IL3000 of *Trypanosoma congolense*, a parasitic kinetoplastid. My re-analysis confirmed these alignments, but surprisingly these proteins hit no other species outside the chordates, not even close relatives of *T*. *congolense*. The three human genes matched only two genes in *T*. *congolense*, accessions CCD16210 and CCD11566.

I aligned these two *T*. *congolense* proteins to all proteins in GenBank, and found that both are mouse proteins, with CCD16210 showing 100 % identity over 184 amino acids and CCD11566 showing 96 % identity over 168 amino acids to mouse proteins. The *T*. *congolense* genome sequence is a draft genome, and these results show clearly that the genes in question derive from contaminants in that assembly. This result provides a more mundane and more probable explanation than HGT for the alignments of these three human genes to *T*. *congolense*.

One of the remaining four genes, ENSG00000212907, is mitochondrially encoded NADH dehydrogenase 4L. As discussed above, mitochondrial genes did indeed result from HGT, when the ancestor of all eukaryotes absorbed the ancient mitochondrion. These genes sometimes have their best hits in bacteria because there is no chromosomal ortholog for some of them, but they do not represent new examples of HGT.

Another of the four genes, ENSG00000242265, is derived from a retrotransposon, as its name (retrotransposon-derived protein PEG10) indicates. Retrotransposons are the result of retroviral integration into the host genome, and thousands of these virus-derived genes have been integrated into the human lineage over time. The vast majority are not functional, but a handful are functional, and virus-to-human HGT events have been widely documented. This explanation is much more plausible than the hypothesis that this gene was horizontally transferred from fungi, as reported in Crisp et al. [1].

This analysis leaves just two genes of the 28 that have an HGT index consistent with Crisp et al.'s. primary criterion for HGT. The first of them, ENSG00000148288 (globoside α-1,3-N-acetylgalactosaminyltransferase 1), matches a bacterial gene from *Parachlamydia acanthamoebae*. It is found in a number of other bacteria and in many vertebrates, but its best alignment to non-chordate metazoans has a non-significant match to *Cimex lectularis*, a bedbug, with bitscore of 37. Interestingly, though, this gene is found in two other very distant species, *C*. *owczarzaki* and *Salpingoeca rosetta*, both in the clade Opisthokonta, which includes both animals and fungi. The matches to these two species have a bitscore of 120, while the best bacterial match has a bitscore of 130. Thus while HGT of this gene cannot be entirely ruled out, one would have to argue that it was also transferred separately into each of these two other, very distant species. This seems highly unlikely; it may instead have simply evolved more rapidly in other clades: the matches described here, with bitscores from 120 to 130, are only 33–35 % identical and have already diverged greatly from the human protein.

The final gene to consider is ENGS00000216937 (*Coiled-coil domain-containing protein 7*). This gene is widely distributed in vertebrates and has distant homologs in plants, protists, and bacteria. Crisp et al. [1] claim it was horizontally transferred from protists, but that hypothesis has no more evidence to support it than a claim of HGT from plants or bacteria. It is much more likely that this gene has simply been lost from the non-chordate genomes whose sequence is available today. An alternative hypothesis, given how distant the alignments are for this gene to non-vertebrates, is that orthologs in other species have evolved slightly faster and are no longer detectable by pairwise alignment.

**Abbreviations {1^st^ level heading}**

BVT, bacterial-vertebrate transfer; *h*, HGT index; HGT, horizontal gene transfer.

**Additional references**

1. Crisp A, Boschetti C, Perry M, Tunnacliffe A, Micklem G. Expression of multiple horizontally acquired genes is a hallmark of both vertebrate and invertebrate genomes. Genome Biol. 2015;16:50.
2. Boschetti C, Carr A, Crisp A, Eyres I, Wang-Koh Y, Lubzens E, et al. Biochemical diversification through foreign gene expression in bdelloid rotifers. PLoS Genet. 2012;8:e1003035.
3. Altschul SF, Madden TL, Schaffer AA, Zhang J, Zhang Z, Miller W, Lipman DJ. Gapped BLAST and PSI-BLAST: a new generation of protein database search programs. Nucleic Acids Res. 1997;25:3389–402.
4. The International Human Genome Sequencing Consortium. Initial sequencing and analysis of the human genome. Nature. 2001;409:860–921.
5. Erwin DH, Davidson EH. The last common bilaterian ancestor. Development. 2002;129:3021–32.
6. Salzberg SL, White O, Peterson J, Eisen JA. Microbial genes in the human genome: lateral transfer or gene loss? Science. 2001;292:1903–6.
7. Stanhope MJ, Lupas A, Italia MJ, Koretke KK, Volker C, Brown JR. Phylogenetic analyses do not support horizontal gene transfers from bacteria to vertebrates. Nature. 2001;411:940–4.
8. Genereux DP, Logsdon JM, Jr. Much ado about bacteria-to-vertebrate lateral gene transfer. Trends Genet. 2003;19:191–5.
9. Nebert DW, Wikvall K, Miller WL. Human cytochromes P450 in health and disease. Philos Trans R Soc Lond B Biol Sci. 2013;368:20120431.
10. Suga H, Chen Z, de Mendoza A, Sebe-Pedros A, Brown MW, Kramer E, et al. The *Capsaspora* genome reveals a complex unicellular prehistory of animals. Nat Commun. 2013;4:2325.
11. Li L, Stoeckert CJ, Jr, Roos DS. OrthoMCL: identification of ortholog groups for eukaryotic genomes. Genome Res. 2003;13:2178–89.
